# Streamlined analytical strategy using resonance Rayleigh scattering signal amplification for nanoscale quantification of rasagiline in tablets with content assessment

**DOI:** 10.1186/s13065-025-01585-0

**Published:** 2025-07-26

**Authors:** Ahmed A. Abu-hassan

**Affiliations:** Department of Pharmaceutical Analytical Chemistry, Faculty of Pharmacy, Assiut Branch, Al-Azhar University, Assiut, 71524 Egypt

**Keywords:** Rasagiline, RRS, Synchronous spectrofluorimetry, Content uniformity, Validation

## Abstract

Rasagiline (RSG), a prevalent drug for Parkinson’s disease, is classified as a monoamine oxidase inhibitor. These drugs operate by elevating dopamine levels in the brain, with the objective of alleviating symptoms related to the illness. This work utilized a sensitive and feasible experimental approach to assess the amount of RSG. An experiment in a single pot, which is compatible with green chemistry, was used. The fundamental premise of this approach was the molecular-size-dependent resonance Rayleigh scattering phenomenon, arising from an association between the dual complex of Erythrosine and RSG. The combination of RSG medicine and Erythrosine in an acidic environment resulted in the formation of an association complex, which amplified the resonance Rayleigh scattering (RRS) signal. The increase in signal was directly associated with the concentration of RSG, particularly within the range of 50-1400 ng/mL. The amplification of the RRS signal was observed at a wavelength of 354 nm. Determining the limit of detection at 15.18 ng/mL and the limit of quantitation at 46 ng/mL demonstrated the method’s sensitivity. The method’s attributes were meticulously examined and refined. The methodological approach was validated in compliance with the International Council for Harmonisation (ICH) requirements to verify its dependability. Moreover, the approach was effectively utilized to assess RSG in its designated dose form. The utilization of existing RRS innovation to assess the target drug was extend to estimate content homogeneity was an impressive accomplishment.

**Clinical trial number**: Not applicable.

## Introduction

Monoamine oxidase (MAO), a critical enzyme present in diverse tissues such as the liver, nervous system, and gastrointestinal tract, is essential for the oxidative deamination of monoamines. These compounds, which include neurotransmitters and dietary amines, may originate endogenously or from external sources [1, 2]. Beyond regulating physiological processes, MAO facilitates the metabolism and elimination of both endogenous and exogenous amines, underscoring its broad functional significance. Clinically, MAO inhibitors have emerged as valuable therapeutic agents, particularly in addressing neurodegenerative and psychiatric conditions such as Parkinson’s disease and depression [3, 4]. The enzyme exists in two isoforms: MAO-A, which primarily degrades serotonin, noradrenaline, and adrenaline, and MAO-B, which metabolizes dopamine, β-phenylethylamine, and benzylamine [5, 6]. This specificity influences therapeutic outcomes, as MAO-A inhibition modulates mood-related neurotransmitters, while MAO-B inhibition elevates dopamine levels—a key target in managing Parkinson’s disease.

Among modern MAO inhibitors, RSG—a second-generation MAO-B inhibitor with the chemical formula (R)-N-2-Propynyl-1-indanamine and a molar mass of 171.243 g·mol⁻¹—has gained prominence. Approved for once-daily oral use in regions such as the United States, European Union, and Canada, RSG serves as both monotherapy and adjunct treatment for Parkinson’s disease. Studies highlight its efficacy in alleviating early-stage symptoms and enhancing motor function in advanced cases when combined with levodopa, notably extending “on” periods (improved mobility) and reducing “off” episodes (symptom reemergence) without inducing severe dyskinesias [7, 8]. Its favorable tolerability profile and dosing simplicity further position RSG as a pragmatic first-line option for early intervention and a complementary therapy in later disease stages. Notably, RSG demonstrates significantly greater potency (5–10-fold) compared to selegiline, another MAO-B inhibitor, enhancing its therapeutic appeal [9]. This introduction explores the biochemical role of MAO, the clinical relevance of its inhibitors, and the distinctive advantages of rasagiline in Parkinson’s disease management.

Literature study revealed a paucity of published analytical methods for RSG measurement in plasma, tablets, and bulk forms across several journals. Certain methodologies encompass spectrophotometric techniques among these processes, the interaction of RSG with tetrachloro-1,4-benzoquinone is used for procedure 1, chloranilic acid for procedure 2, and 7,7,8,8-tetracyanoquinodimethane for procedure 3. The generated colored products were quantified spectrophotometrically at 535, 524, and 843 nm for procedures 1, 2, and 3, respectively [10]. Another process includes diazotizing sulphanilic acid in acidic circumstances with sodium nitrite, then combining it with RSG in an alkaline medium. The yellow color created by RSG and the positive diazonium ion is strongest at a wavelength of 440 nm [11]. Multivariate calibration strategy for the UV spectrophotometric assay of RSG in bulk drugs and dosage formulations was conducted at 215 nm [12]. A new way has been developed to create colored ion-pair complexes of the RSG drug using bromocresol green, bromothymol blue, and bromophenol blue in an acidic solution, which can then be extracted using chloroform. The extracted colored complexes showed absorbance at maxima of 414 nm for all three strategies [13]. Different HPLC approaches were established for RSG assay using acetonitrile and phosphate buffer (50:50 v/v) as a mobile phase. The flow rate was 0.5 ml/min, and 210 nm was the wavelength for detection [14]. RP-HPLC strategy was developed as a stability-indicating approach for the concurrent estimation of RSG-related degradation and impurities [15]. Another RP-HPLC with UV detection at 210 nm was used for RSG tablets estimation [16]. HPLC technique utilizing fluorescence detection for the quantification of RSG and its uses in medicinal formulations [17]. RSG was isolated using an LLE procedure and subsequently quantified via RP-HPLC. The mobile phase was a mix of ammonium acetate buffer (set to pH 5.8) and acetonitrile in a 55:45 ratio, flowing steadily at 1.0 mL/min. The separation of the substances was done using a Lichrosphere RP-C18 analytical column, and a UV detector was used to identify them at a wavelength of 265 nm [18]. Separation of RSG was achieved using silica gel 60 F254 thin-layer chromatography plates with a mobile phase composed of butanol, methanol, and water in a 6:1:2 volumetric ratio. This yielded well-defined spots with a retention factor (Rf) of 0.76 ± 0.01. Quantitative evaluation was conducted via densitometric scanning at a wavelength of 255 nm [19]. A novel square wave voltammetry approach was established and validated for quantifying RSG in bulk, Tablets, and human plasma. This method leverages the cathodic activity of the compound’s acetylene group detected via hanging mercury drop electrode [20]. The detection of RSG by spectrofluorimetry were by reaction with NBD-Cl [21].

A molecular-size-selective RRS analytical platform was engineered to enable high-sensitivity pharmaceutical analysis, utilizing xanthene-based chromophores with distinct structural and spectral properties as molecular probes. This methodology exploits the formation of ion-association complexes between target analytes and tunable dye systems, where certain xanthene derivatives exhibit inherent fluorescence due to their rigid, macrocyclic architectures. pH-modulated ionization of these dyes facilitates selective electrostatic interactions with analytes, enabling size-dependent signal amplification through complexation. The system demonstrates broad applicability for drug quantification in complex matrices, leveraging the synergistic effects of molecular size discrimination and chromophoric resonance enhancement [22]. Structural or electronic modifications to molecular probes can induce distinct physicochemical responses, including chromogenic transformation [23], quenching of intrinsic fluorescence [24, 25], or amplification of resonance Rayleigh scattering (RRS) intensity [26, 27]. These measurable spectral shifts enable precise quantification of analytes through selective interactions. For ultra-trace pharmaceutical detection (ng/mL range), RRS-based methodologies are particularly advantageous due to their operational simplicity, non-destructive nature, and exceptional sensitivity. The technique’s versatility is evidenced by its application across diverse domains, such as biomolecular analysis (e.g., nucleic acids [28], glycosaminoglycans [29], and proteins [30]), pharmacokinetic profiling [31], environmental monitoring (heavy metal ions [32]), and organic contaminant detection [33]. This broad utility stems from the technique’s ability to transduce molecular recognition events into quantifiable optical signals with minimal sample preparation. Beyond molecular probes, advanced materials like metal-organic frameworks (MOFs) and covalent organic frameworks (COFs) show promise in drug sensing. For example, luminescent COFs enable multi-analyte detection, while MOFs facilitate pesticide/drug removal. Though not employed here, such materials highlight evolving strategies for trace analysis [34–36].

Erythrosine, a xanthene-derived chromophore, serves as a critical analytical tool in pharmaceutical quantification due to its selective interaction with drug molecules. Under acidic conditions, the secondary amine group of RSG undergoes protonation, enabling electrostatic complexation with the anionic Erythrosine dye. This association generates a dual-mode amplification system (“on-on” fluorescence coupled with resonance Rayleigh scattering, RRS), where the RRS signal intensification exhibits a linear correlation with RSG concentration. The methodology demonstrates economic viability for routine quality control applications, owing to its reliance on low-cost reagents and compatibility with standardized laboratory instrumentation. Notably, the system’s analytical sensitivity is augmented by the dye’s intrinsic spectral properties, which are modulated by structural interactions during ion-pair formation, rendering it advantageous for trace-level drug monitoring in resource-efficient settings.

## Experimental

### Apparatus

Fluorescence emission spectra were acquired using a SCINCO spectrometer (Korea) equipped with a 150 W xenon arc lamp excitation source in a single-cell configuration. The photomultiplier tube (PMT) detector operated at a scan speed of 570 nm/min with an integration time of 50 ms. Sample solutions were homogenized using a SC-101TH ultrasonic bath sonicator (Sonicor). The pH of the solutions was calibrated with a pH700 benchtop meter (Eutech Instruments, Singapore), ensuring precise adjustments.

### Materials and reagents

Dopaminect^®^ tablets (1.0 mg per tablet) were procured from El Ezaby Pharmacy, Egypt. The rasagiline active compound was sourced from Marcyrl Co., Egypt, a specialty chemical manufacturer. For spectroscopic analysis, erythrosine dye (SigmaPure Laboratories) was prepared by homogenizing 20 mg of the compound with 20 mL of deionized water using a vortex mixer. This stock solution was subsequently diluted to 100 mL with water, yielding a final concentration of 0.02% (w/v). High-purity organic solvents were supplied by TantaChem Supplies, a distributor based in Tanta, Egypt. Key reagents—citric acid, hydrochloric acid (HCl), phosphoric acid, and sodium hydroxide (NaOH)—were obtained from Egypt’s National Chemical Industries. A 0.1 M Torell buffer system was formulated by blending precise ratios of NaOH, H_3_PO_4_, citric acid, and HCl, followed by pH calibration to the required value using a digital pH analyzer.

### Preparation of stock solutions

A standardized RSG stock solution was formulated by dissolving 25 mg of the active RSG pharmaceutical ingredient in deionized water, followed by transfer to a 250 mL volumetric flask for precise volume adjustment. Working concentrations were subsequently prepared through systematic dilution of the stock solution with ultrapure water. To ensure chemical integrity, all stock solutions were stored at 4 °C in a temperature-controlled environment throughout the experimental workflow.

### Calibration protocol and measurement specifications

In the RRS assay, measured volumes of RSG within an analytical range of 0.5–14 µg/mL were introduced into 10 mL calibrated glass tubes. Each sample received 1.2 mL of torell-buffered solution (pH 3.6) and 1.2 mL of erythrosine dye. Following a 5-minute incubation period at ambient temperature, the mixtures were diluted with ultrapure water and vortex-mixed briefly for homogenization. A reagent blank, prepared identically but excluding RSG, served as the control. The RRS signal amplification induced by RSG-Dye complexation was quantified at 354 nm using a spectrofluorometer. A calibration graph correlating ΔIRRS (net scattering intensity) to RSG concentration was constructed for quantitative analysis.

ΔIRRS represents the differential scattering intensity between the RSG-Dye complex and the baseline signal of unbound dye. The enhanced RRS signal arises from molecular interactions between RSG and erythrosine, enabling the calculation of drug concentration via the relationship:

ΔIRRS = IRRS (complex) − I0,RRS (dye alone).

### Analysis of dosage forms

Fifteen Dopaminect tablets (rasagiline mesylate formulation) were individually weighed and pulverized to a uniform powder. An aliquot equivalent to 5 mg of RSG was placed in a volumetric flask with a capacity of 50 mL, subsequently followed by the incorporation of 20 mL of deionized water. The suspension underwent ultrasonic agitation for 16 min using a bath sonicator, after which the volume was adjusted to the flask’s calibration mark with water. The content was filtered through a 0.45 μm membrane filter, discarding the initial 10 mL of filtrate to eliminate particulate interference. A 2 mL portion of the clarified filtrate was diluted to 10 mL with aqueous solvent to align with the assay’s linear dynamic range.

For tablet uniformity assessment, individual tablets were separately processed: each was crushed, dissolved in 5 mL of water via sonication, filtered, and diluted to 10 mL. Calibration standards and samples were treated identically, with scattering intensity measurements performed at 354 nm to quantify RSG content relative to the established calibration curve.

## Results and discussion

**Fig. 1:**
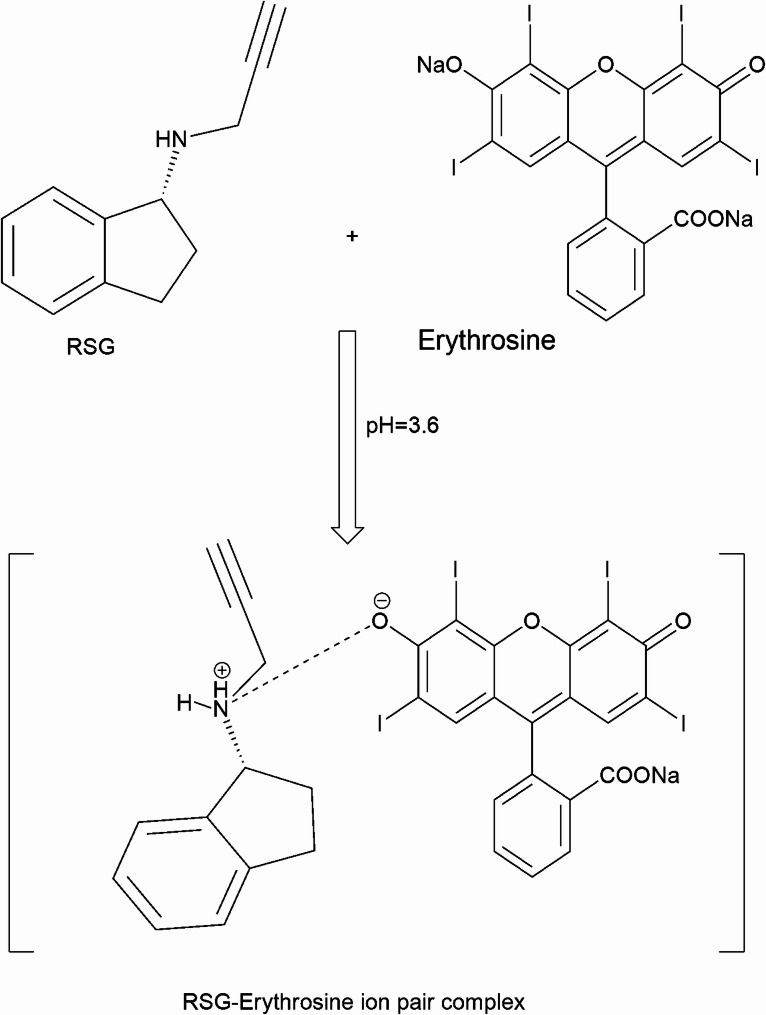
Representation for association complex formation between RSG and Erythrosine

**Fig. 2:**
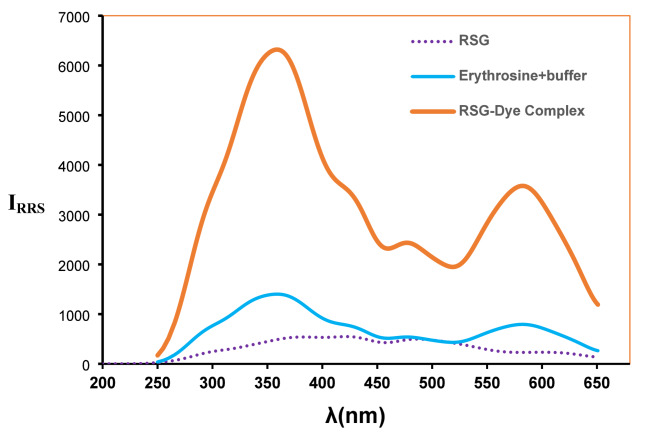
RRS spectrum of, RSG, and the formed RSG- Erythrosine association complex

Xanthene-based dyes have established themselves as integral tools in spectroscopic research, distinguished by their robust intrinsic fluorescence and ability to form stable complexes with a variety of pharmacologically active compounds. These dyes, including fluorescein, eosin, and erythrosine, enable the application of diverse analytical techniques such as colorimetry [23], spectrofluorometry [24, 25], and resonance Rayleigh scattering [26, 27] (RRS). Their interactions with target molecules often yield distinctive spectroscopic phenomena, including the generation of novel chromogenic responses, quenching of native fluorescence, and significant enhancement of RRS signal intensity. In the current study, erythrosine was employed as a key component in an RRS-based analytical strategy for the detection and quantification of RSG. Under acidic conditions, protonation of the secondary amino group in RSG facilitates electrostatic interaction with erythrosine, leading to the formation of a stable ion-pair complex (Fig. 1). This association generates a synergistic “on-on” fluorescence-RRS dual-response system, wherein the amplified RRS signal exhibits a linear relationship with RSG concentration, as illustrated in Fig. 2. The method’s practicality is underscored by its operational safety and cost-effectiveness, driven by the use of economically accessible reagents and reliance on instrumentation routinely available in quality control laboratories. This combination of analytical precision and logistical feasibility positions the approach as a viable solution for routine pharmaceutical analysis.

### Adjusting the experimental variables

Validating and adjusting the different procedural variables anticipated to affect the observed response was crucial for achieving optimal results with the suggested spectroscopic method. In line with this, various experimental conditions were systematically tested and assessed to enhance the recorded response.

#### Impact of pH and buffer amount

**Fig. 3:**
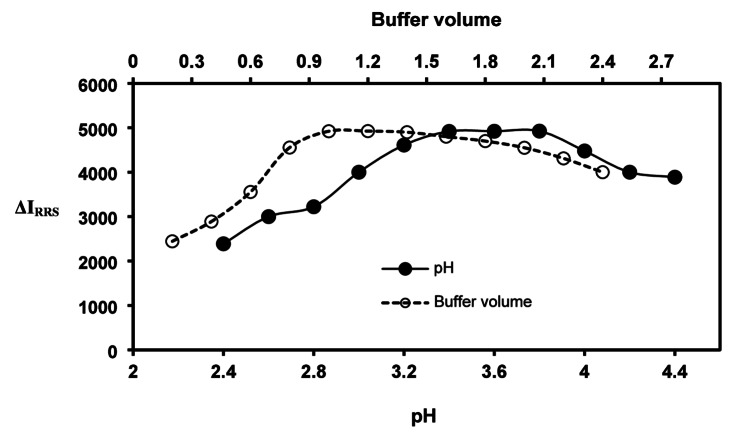
Impact of buffer volume and pH on the RRS of the current system

The impact of pH on the process of complexation of RSG with erythrosine was examined across a pH range of 2.4 to 4.4. Figure 3 clearly illustrates that the level of ΔIRRS peaked at pH 3.6 and subsequently declined when deviating from this optimal point. Furthermore, the impact of buffer volume on the RRS signal was examined across a range of 0.2 to 2.4 mL of Torell buffer. The optimum reaction was attained with 1.2 ml of buffer, while increased buffer volumes resulted in diminished responses due to cationic competition, and smaller volumes were insufficient to maintain the pH value.

#### Volume of erythrosine reagent

**Fig. 4:**
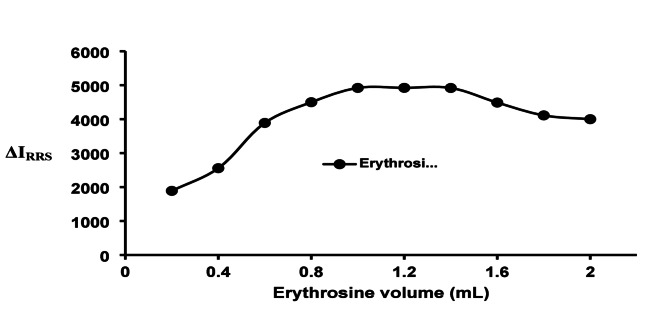
Impact of various volumes of Erythrosine (0.02 W/v) on the proposed RRS strategy

In the present study, the erythrosine reagent was employed at different concentrations. A master solution of erythrosine (0.02 W/V) was furnished, and various volumes (from 0.2 to 2.0 mL) of this solution were utilized in the assay. Following each experiment, the value of the RRS signal was documented. Smaller volumes of the master solution (0.2–0.8 mL) were noted to progressively enhance the RRS signal, with the peak RRS response occurring within the volume range of 1.0 to 1.4 (Fig. 4). Following this point, a decline in RRS signal was observed, potentially linked to the propensity of erythrosine to self-aggregate.

#### Dispersing solvent and time impact

**Fig. 5:**
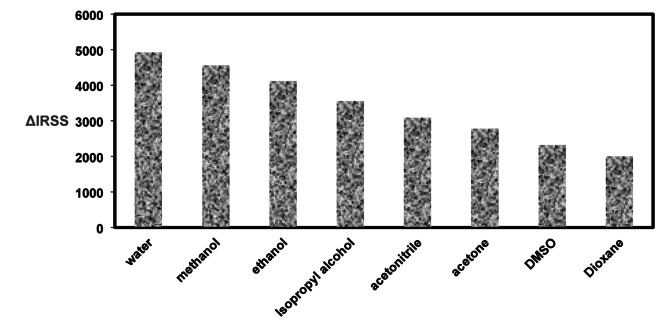
Illustrating the impact of the variety of diluting solvents on RRS of the current strategy

The investigation involved testing different dispersing mediums, such as water, various alcohols (isopropyl, ethyl, methyl), acetonitrile, DMSO, acetone, and Dioxane. The application of water produced the most favorable outcomes regarding the enhancement of RRS (Fig. 5**).** The use of water, recognized for its properties as a green solvent with a notable polarity index of approximately 9 and a dielectric constant of 80.2, significantly aided in the efficient formation of the RSG-Dye association complex. The suboptimal results noted with alternative organic solvents could be linked to their ability to compromise the integrity of the final complex. The potential of these solvents to alter or denature the stability of the complex system is noteworthy. The selected system demonstrates complete miscibility in water, attributable to the solubility of its components in the aqueous medium. Nonetheless, low miscibility can arise with various organic solvents as a result of disparities in their dielectric constants, potentially complicating the formation of the complex system.

### Method validation

The ICH guidelines were followed to validate the method. Several performance characteristics, including linearity, precision, approach range, accuracy, robustness, LOD, and LOQ, are used in the validation process of the invented spectrofluorimetric approach.

#### Linearity and concentration range

**Fig. 6:**
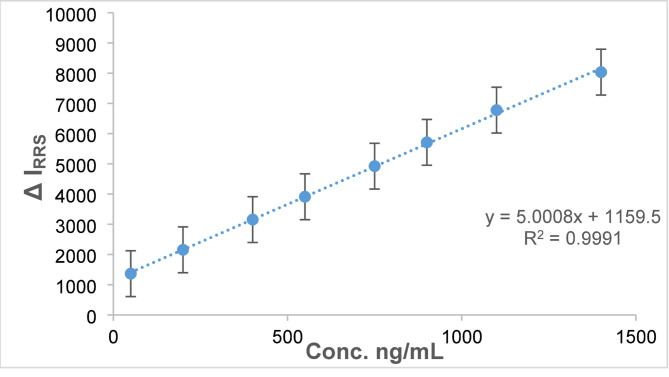
Calibration curve of the proposed spectroscopic method

The RRS examination method that was developed was employed to examine standard RSG solutions. A calibration graph was developed by making a plot of the distinction in RRS against RSG concentrations in ng/ml. The RRS methodology demonstrated a linear response across a concentration range of 50-1400 ng/mL, exhibiting impressive correlation coefficients (Fig. 6). The calibration was represented by a regression equation (Y = 5 X + 1159.5, *r* = 0.9995), with the relevant data detailed in Table 1. The analysis of sensitivity parameters, including the Limit of Quantification (LOQ) and the Limit of Detection (LOD), was conducted using the equations established by the International Council on Harmonisation (ICH). The technique demonstrated a sophisticated degree of sensitivity because of the high molecular weights of the dye and the therapeutic component.

**Table 1:** Information on the developed system’s statistics

Parameters	Values
Linear range (ng/mL)	50-1400
Slope	5
Intercept	1159.5
SD of intercept (S_a_)	23
Correlation coefficient (r)	0.9995
Determination coefficients (r^2^)	0.9991
Number of determinations	8
Limit of quantitation (ng/mL)	46
Limit of detection (ng/mL)	15.18

#### Accuracy and precision

Investigations were performed on the suggested approach to evaluate its spectroscopic accuracy. Four distinct content levels (200, 550, 900, and 1200 ng/mL) were employed in the process. The RRS spectroscopic measurement method was assessed for accuracy through the use of percentage error and recuperation percentage metrics. Table 2 presents the results that confirm the accuracy of the technique. The established methodology was utilized to analyze standard drug materials at three distinct doses (300, 750, and 1100 ng/mL) to assess inter-day and intra-day precision. The parameter of the proposed RRS strategy was assessed through the calculation of the Relative Average Standard Deviation (RSD). The RSD values at low and middle levels were reasonable, remaining below 2%, as indicated in Table 3.

**Table 2:** Accuracy of the developed approach at four RSG concentrations

Conc. Level (ng/mL)	Recovery^*^± SD	% Error
200	98.88 ± 0.76	1.12
550	100.68 ± 0.60	0.68
900	101.79 ± 1.76	1.79
1200	98.81 ± 1.39	1.19

*mean of three RSG determinations and SD is the standard deviation

**Table 3:** Precision of the current strategy at two levels

Conc. Level ( ng/mL )	Intraday precision		Interday precision	
300	101.12 ± 1.40	1.38	99.14 ± 1.68	1.69
750	98.86 ± 1.11	1.12	98.52 ± 0.63	0.64
1100	100.92 ± 1.31	1.30	101.21 ± 1.99	1.97

*mean of three RSG determinations and RSD is the relative standard deviation

#### Robustness

The durability of the RRS strategy was assessed by examining the system’s performance in response to minor alterations in key laboratory variables, such as pH, buffer volume, and dye amount. Minor variations in the aforementioned variables were found to have little impact on the ΔIRRS values, as indicated by the deviations from the mean and recovery% presented in Table 4. Thus, the method demonstrated robustness, as minor procedural variations remained within the accuracy appreciation of the developed approach.

**Table 4:** Evaluating the robustness of the existing RSG assay technology

Parameter	± optimum value	% Recovery* ± SD
pH	3.5	99.05 ± 1.37
	3.7	98.25 ± 1.06
Buffer volume (mL)	1.1	98.24 ± 0.86
	1.3	99.24 ± 0.75
Dye volume (mL)	1.1	101.43 ± 0.84
	1.3	100.30 ± 1.06

*Mean of three replicate measurements, SD = Standard deviation

#### Selectivity

To confirm method selectivity, common excipients (glucose, lactose, flour, talc, magnesium stearate, sorbitol) were evaluated. Table 5 demonstrates their lack of interference, with recoveries near 100% and minimal standard deviation. Since these molecules lack the requisite amino group, they cannot form the RSG-erythrosine complex responsible for the signal.

**Table 5:** Selectivity assessment of the proposed method

Excipients	Amount added (µg mL^− 1^)	RRS method Added drug con. (750 ng mL- ^1^) % Recovery ± SD^*^
Mg stearate	10	
Starch	40	
Lactose	10	
Glucose	10	
Talc	10	
Sorbitol	10	

*Mean of three RSG determinations

## Application of the suggested methodology

The recent study evaluated the composition of (RSG)-based pharmaceutical products, including the branded formulations, Parkintreat tablets, available in Egyptian pharmacies. The newly developed analytical method was employed to assess drug content and compare its performance with an established spectroscopic technique described in reference [21]. Both methods were applied to analyze formulations of identical dosage strengths. To evaluate equivalence, recovery percentages from the two approaches were statistically compared using F-tests (to assess variance in precision) and Student’s t-tests (to evaluate mean accuracy). The calculated F and t values for all tested formulations fell below their respective critical thresholds at a 95% confidence level, indicating no statistically significant differences in precision or accuracy between the novel method and the reference spectroscopic protocol. These findings confirm that the newly developed process meets the same rigorous quality standards as the published strategy, demonstrating its suitability for pharmaceutical quality control of RSG products. The results, summarized in Table 6, highlight the method’s reliability for ensuring consistent RSG drug content in commercial anti-Parkinsonism medications within regional markets.

**Table 6:** Dosage form analysis of RSG and comparison with the reported method

Dosage form	Current method	Reported method		
	Recovery* ± SD	Recovery* ± SD	t- test value^a^	F-value^a^
Parkintreat^**R**^**tablets**	98.64 ± 1.63	98.37 ± 1.01	0.32	2.60

^*^ The value is the average of five determinations for both the proposed and reported methods

^a^ Tabulated values at 95% confidence limit are t = 2.306, F = 6.338

### Testing of content uniformity

Homogenization checks were conducted on the content of each tablet under USP guidelines; specifically, ten Parkintreat tablets were analyzed individually. Following the transfer of the powder from a single tablet to a volumetric flask, sonication was performed for 16 min, and recovery percentage tests were conducted on each tablet to quantify RSG variability. The results demonstrated that the RSG concentration in the analyzed tablets is homogeneous, thereby ensuring the dependability of the suggested strategy for determining content homogeneity (Table 7**).** Finally, a tabular form for comparison between the proposed method and some reported methods has been inserted in Table 8.

**Table 7:** Checking content uniformity of Parkintreat^R^ tablets by the current approach

Capsule number	%Recovery
1	98.24
2	97.32
3	96.01
4	99.04
5	101.80
6	102.70
7	98.98
8	97.16
9	98.68
10	98.25
Mean ($\:\overline{X}$) ± S.D	98.82 ± 2.04
Acceptance value (AV)	4.90
Max. allowed AV (L1)	15

**Table 8:** A comparison between the proposed method and some reported methods

Methods	Reagent	Linear range	LOQ	LOD	Application
**Proposed method**	Erythrosine	50–1400 ng/mL	46 ng/mL	15.18 ng/mL	Tablets/content uniformity
**Reported method **[10]	chloranilic acid for method A, tetrachloro-1,4-benzoquinone for method B, and 7,7,8,8-tetracyanoquinodimethane for method C.	25– 300 𝜇g mL^−1^, 25–350 𝜇g mL^−1^, and 50–500 𝜇g mL^−1^ for methods A, B, and C, respectively.	0.407, 0.932, and 1.94 𝜇g mL^−1^ for methods A, B, and C, respectively.	0.122, 0.279, 0.583 𝜇g mL^−1^ for methods A, B, and C, respectively.	Tablets
**Reported method** [11]	Diazotization of sulphanilic acid under acidic conditions in presence of sodium nitrite.	0–10 µg mL^− 1^	0.1 µg mL^− 1^	0.033 µg mL^− 1^	Authentic powder
**Reported method** [13]	bromothymol blue, bromophenol blue, and bromocresol green in acidic medium.	3.0–30, 3.0.0–30 and 2.0–25 µg/ml	1.23, 1.99, and 0.57 µg/ml	0.4, 0.66, and 0.19 µg/ml	Tablets
**Reported method** [21]	NBD-Cl	30–600 ng/ml	27 ng/ml	8.9 ng/ml	Tablets/content uniformity/plasma

## Estimation of the binding constant and calculation of gibbs free energy

The coupling constant (K) between the dye and drug was determined using the Benesi-modified Hildebrand equation [37]. This equation is expressed as: 1/ΔR = 1/ΔR_max_ + 1/(K[D]ΔR_max_). In this formulation, ΔR represents the difference in RRS strength (R– R₀), where R₀ is the emission of the blank solution, F is the RRS at a given RSG concentration, and R_max_ is the RRS at full saturation. Consequently, ΔR_max_ is defined as R_max_– R₀. The equation incorporates the binding constant (K) and the RSG concentration ([D]), with [D] expressed in molarity.

**Fig. 7:**
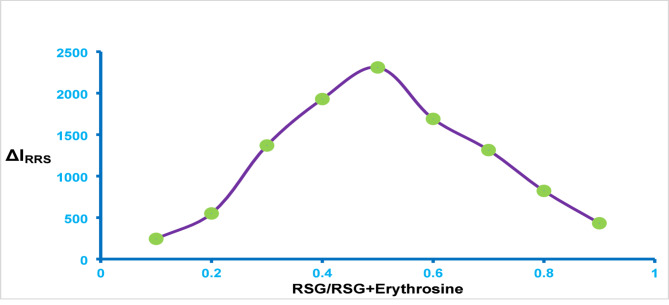
Job‘s plot of continuous variation for the complex formation between erythrosine B and RSG

A linear relationship was established by plotting 1/[D] on the x-axis against 1/ΔR on the y-axis. The binding constant (K) was then derived from the slope and intercept of the resulting straight line. The calculated coupling constant was 8.3 × 10^4^. The standard free energy change (ΔG°) for the interaction was calculated using the formula ΔG° = -2.303 RT log K, consistent with the method described in reference [38]. Here, R denotes the universal gas constant, T is the absolute temperature in Kelvin, and K is the binding constant determined previously. The calculated Gibbs free energy for the reaction was − 28.06 kJ mol⁻¹. This negative value indicates that the reaction is highly spontaneous at ambient temperature. Finally, the stoichiometry of the RSG-dye complex was determined via the Job’s plots method. Equimolar solutions of the dye and the medication were formulated and integrated into the overall method, utilising complementary volumes of each solution. The RRS was measured for each solution, and the increase in RRS at 354 nm was plotted against the drug mole fraction. A maximum value was obtained in the plot at a drug mole fraction of about 0.5 (Fig. 7). This value confirmed the formation of a binary complex that contains drug: dye in a ratio of 1:1.

## Conclusion

This established study presents a novel method for quantifying RSG. The method demonstrates sensitivity and simplicity, rendering it a significant asset in research. RSG concentrations between 50 and 1400 ng/mL were identified as an ion pair via electrostatic attraction in a mildly acidic environment. The application of the erythrosine reagent presents limited risks in comparison to alternative methods. This method offers advantages such as simplicity, environmental sustainability, and straightforward RSG quantification in aqueous solutions. This approach utilizes eco-friendly techniques that do not involve evaporable liquids. The method effectively identified RSG in various forms, including both authentic pharmaceutical products and assessments of homogeneity.

## Data Availability

The data will be available upon reasonable request from the corresponding author.

## Abbreviations

HPLC: High-performance liquid chromatography
ICH: International council for harmonisation
LOD: Limit of detection
LOQ: Limit of quantitation
MAO: Monoamine oxidase
NBD-Cl: 4-Chloro-7-nitrobenzofurazan
PMT: Photomultiplier tube
RRS: Resonance rayleigh scattering
RSD: Relative standard deviation
RSG: Rasagiline
TLC: Thin-layer chromatography
USP: United states pharmacopeia
